# Interaction between sleep duration and physical activity on mortality among cancer survivors: findings from National Health and Nutrition Examination Surveys 2007–2018

**DOI:** 10.3389/fpubh.2025.1532320

**Published:** 2025-01-17

**Authors:** Ruyan Chen, Jianglong Han, Si Li, Haiyu Deng, Tingting Jian, Zheyu Huang, Yuxuan Wei, Zhenming Fu

**Affiliations:** Cancer Center, Renmin Hospital of Wuhan University, Wuhan, China

**Keywords:** sleep duration, physical activity, cancer, mortality, National Health and Nutrition Examination Survey (NHANES)

## Abstract

**Background:**

Sleep duration and physical activity (PA) are critical factors influencing mortality risk. However, the interaction between sleep duration and PA with mortality risk among cancer survivors has not been well explored.

**Methods:**

This cross-sectional study utilized data from the National Health and Nutrition Examination Survey (NHANES) spanning 2007–2018. Multivariable Cox regression analysis and restricted cubic splines were employed to evaluate the hazard ratios (HRs) and 95% confidence intervals (CIs) of the association of sleep duration and PA with mortality risk in cancer survivors. Multiplicative and additive interaction terms were constructed to assess interaction effects.

**Results:**

The study included a total of 2,528 adult cancer survivors (aged≥20 years). Sleep duration exhibited a U-shaped association with all-cause and cancer-specific mortality, while demonstrating an inverted L-shaped association with cardiovascular disease (CVD) mortality. Compared to physically inactive participants, those with adequate PA had lower risks of all-cause mortality (HR = 0.542, 95% CI: 0.540–0.543), cancer mortality (HR = 0.486, 95% CI: 0.484–0.488), and CVD mortality (HR = 0.759, 95% CI: 0.755–0.763) among cancer survivors. A significant additive interaction was found between extreme sleep duration and PA on all-cause mortality risk among cancer survivors (long sleep duration: relative excess risk due to interaction (RERI) = 1.514, 95% CI: 1.504–1.525; short sleep duration: RERI = 0.725, 95% CI: 0.713–0.737).

**Conclusion:**

Extreme sleep duration and lack of PA were associated with mortality risk in cancer survivors independently and jointly. Maintain appropriate sleep duration and doing regular PA may synergistically improve cancer survival among cancer survivors.

## 1 Introduction

Cancer remains the leading cause of death globally. In 2020, there were approximately 19.3 million new cancer cases and about 10 million cancer-related deaths worldwide (1). Despite advances in cancer treatment, survivors often face long-term health challenges. Addressing modifiable lifestyle factors, such as sleep and physical activity (PA), is critical for improving quality of life and overall survival in this vulnerable population.

Sleep is a critical determinant of health that is often disrupted in cancer survivors. Cancer survivors experience persistent sleep problems (e.g., short sleep duration) further because of cancer and cancer treatment (2) and may reduce cancer survival (3, 4). Insomnia is characterized by difficulty falling asleep, maintaining sleep or nonrestorative sleep coupled with daytime impairment and occurs at least three times per week for at least 1 month, which can be short-term (<3 months) or chronic (>3 months) (5). Short sleep duration is associated with metabolic dysfunction and immune suppression (6, 7). American Academy of Sleep Medicine (AASM) and Sleep Research Society (SRS) recommend that adults obtain at least 7 h of sleep per night on a regular basis to promote optimal health (8). Moreover, long sleep duration exceeding 9 h per night may also lead to metabolic dysregulation and systemic inflammation (9, 10), adversely impacting the health outcomes of cancer survivors. Dysregulation of circadian rhythms may contribute to the development and progression of cancer (11, 12). A growing body of evidence has supported that both short and long sleep duration was associated with increased mortality among cancer survivors (3, 4).

PA, the major component of life factors, is another important measure affecting cancer survivorship (13). PA can modulate in the immune system and inflammation responses, imparting an antitumorigenic effect (14). Numerous studies support that regular PA was significantly inversely associated with mortality among cancer survivors (15). And there is substantial evidence indicating that PA can be used to manage cancer treatment-related side effects, such as anxiety, depression, impaired physical function, and lymphedema (16–18). Physical inactivity may lead to deconditioning and impaired cardiorespiratory fitness in cancer survivors, increasing the risk of cardiovascular disease (CVD) morbidity and mortality (19). World Health Organization (WHO) recommend that cancer survivors should participate in at least 150 min of moderate PA (e.g., brisk walking, light swimming) or 75 min of vigorous PA (e.g., jogging, running) each week (20). PA is generally safe for cancer survivors (19), however, it is important for survivors to seek medical evaluation prior to starting an exercise program to ensure safety (16).

Systemic inflammation is an important mechanistic pathway for cancer development and progression (21). Inflammation is linked to sleep duration, an increase in circulating inflammation markers such as interleukins (ILs), C-reactive protein (CRP), and tumor necrosis factor-alpha (TNF-*α*) have been found in extremely sleep duration (22). While, regular PA have a potent anti-inflammatory influence. During exercise, the increased release of skeletal muscle-derived IL-6 inhibits the release of pro-inflammatory factors, triggering an anti-inflammatory cascade (23). Sleep duration and PA affect the risk of mortality in cancer survivors through common biological pathways, while PA and sleep are interrelated, but the interaction between sleep duration and PA in mortality risk among cancer survivors has not been elucidated. Although previous studies reported that adequate PA may mitigate the adverse effects of both short and long sleep duration on all-cause and cause-specific mortality in general populations (24, 25), there have been few studies exploring the combined interaction between sleep duration and PA in the risk of mortality among cancer survivors.

This study aims to fill the knowledge gap by investigating the independent associations of sleep duration and PA with the risk of mortality among cancer survivors, as well as how these factors interact to play a role in the risk of mortality. In the study, we analyzed data from the National Health and Nutrition Examination Survey (NHANES) collected between 2007 and 2018 to examine independent associations of sleep duration and PA with mortality in cancer survivors, as well as how these factors interact, offering evidence that can inform targeted interventions and guidelines for improving long-term health outcomes in this vulnerable population.

## 2 Materials and methods

### 2.1 Study population

**Figure 1:**
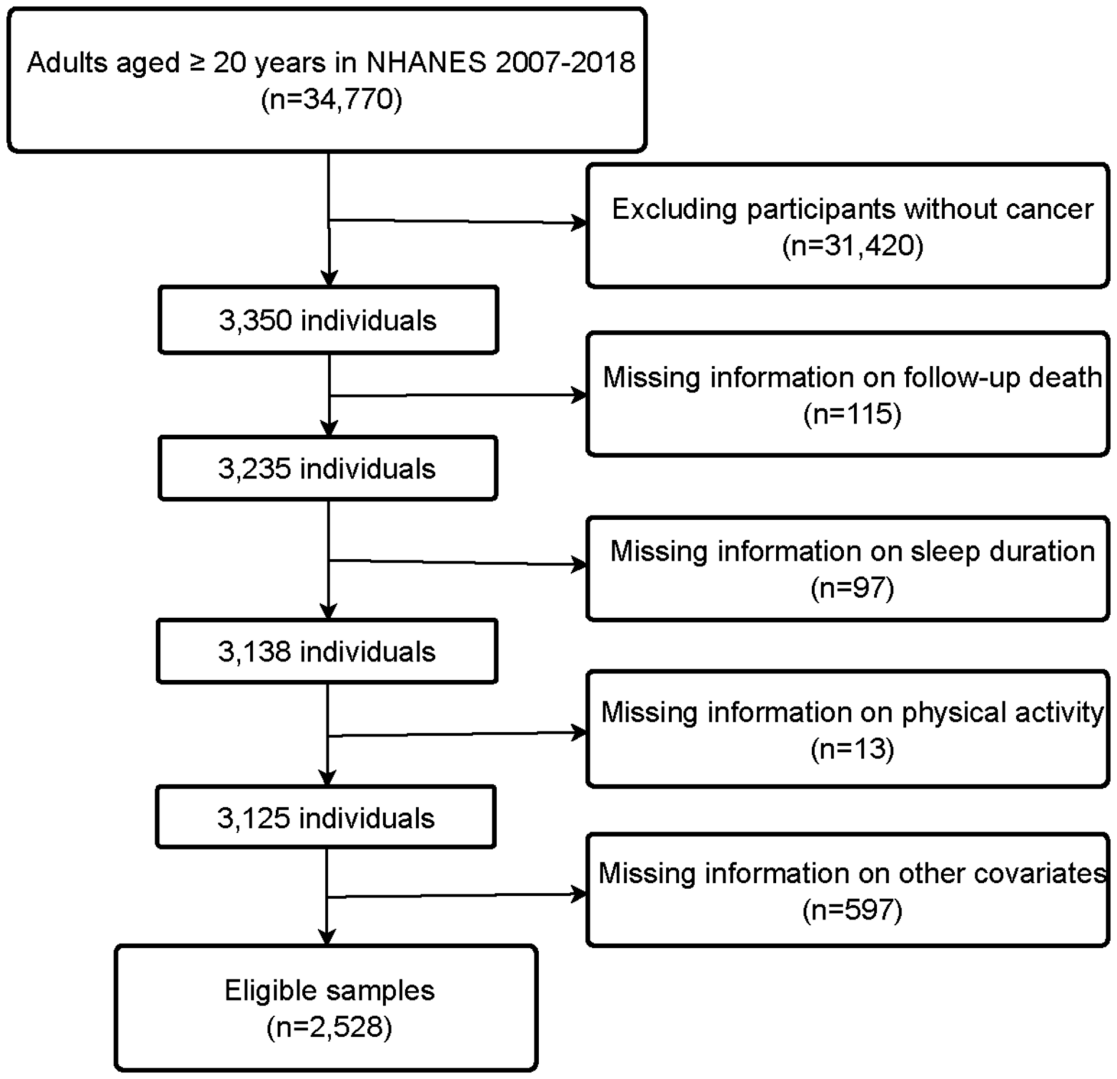
Flowchart of the systematic selection process.

NHANES is a research program managed by the National Center for Health Statistics (NCHS), designed to assess the health and nutritional status of adults and children in the United States (26). All NHANES protocols were approved by the NCHS Ethics Review Board, and written informed consent was obtained from all participants. Additionally, weights are created in NHANES to account for the complex survey design, survey non-response, and post-stratification adjustment to match total population counts from the Census Bureau. We combined data from six consecutive NHANES cycles spanning from 2007 to 2018. Of 34,770 participants extracted from the NHANES database, we excluded those without cancer (*n* = 31,420), as well as those with missing information on follow-up (*n* = 115), sleep duration (*n* = 97), PA (*n* = 13), and other covariates (*n* = 597). Finally, a total of 2,528 participants were included in this study. The flow chart for the selection of study participants is shown in Figure 1.

### 2.2 Diagnosis of cancer

Cancer diagnosis in this study was determined based on the following question: “Have you ever been told by a doctor or other health professional that you had cancer or a malignancy of any kind?” Participants who answered “yes” were classified as cancer survivors.

### 2.3 Assessment of sleep duration

Sleep duration was self-reported by participants. From 2007 to 2016, sleep duration was determined by question: “How much sleep do you usually get at night on weekdays or workdays?” In 2017–2018, participants were asked: (1) “What time do you usually go to sleep on weekdays or workdays?” and (2) “What time do you usually wake up on weekdays or workdays?” Sleep duration was calculated based on these two questions. Sleep duration was further categorized into three groups: short sleep duration (<6 h/night), normal sleep duration (6–8 h/night), and long sleep duration (>8 h/night) (25).

### 2.4 Assessment of physical activity

PA data were obtained using the Global Physical Activity Questionnaire created by the World Health Organization (WHO). PA was converted into weekly metabolic equivalent (MET) minutes of moderate-to-vigorous physical activity (MVPA) using the formula: PA (MET-min/wk) = MET × frequency of PA per week × duration of PA per session. According to the Physical Activity Guidelines for Americans (27), PA was categorized into two groups: Meet MVPA recommendations (≥600 MET-min/wk) or Not meet MVPA recommendations (<600 MET-min/wk).

### 2.5 Ascertainment of mortality

Follow-up data were available up to December 31, 2019, from restricted-use linked mortality files provided by NCHS (28). The causes of death were coded according to the International Classification of Diseases, 10th revision. Cancer mortality was classified as deaths caused by malignant neoplasms (019–043), and cardiovascular disease (CVD) mortality was classified as deaths caused by diseases of the heart (054–064) and cerebrovascular diseases (070).

### 2.6 Assessment of covariates

Demographic information and lifestyle factors were collected through interviews, including age, sex, race, education, smoking status, alcohol consumption, height, weight, sleep disorder and general health condition. Body mass index (BMI) was calculated as weight (kilogram) divided by height (meter) squared. Smokers were defined as participants who had smoked at least 100 cigarettes in their lifetime. Alcohol drinkers were defined as those who had consumed at least 12 drinks in any given year. Sleep disorders were identified by asking participants: “Have you ever told a doctor or other health professional that you have trouble sleeping?” Comorbidities were identified by asking participants: “Has a doctor or health professional ever told you that you have (diabetes/hypertension/CVD).”

### 2.7 Statistical analysis

Baseline characteristics of participants were described according to sleep duration categories. Continuous variables were expressed as weighted means ± standard errors, and categorical variables as counts (weighted percentages). Statistical differences in continuous and categorical variables were assessed using t-tests and chi-square tests, respectively. Multivariable Cox proportional hazards regression models were used to estimate hazard ratios (HRs) and 95% confidence intervals (CIs) for the association of sleep duration and PA with all-cause, cancer, and CVD mortality. Baseline variables were included in the multivariable Cox regression model if they showed *p*-value<0.1 in univariate cox analysis or were clinically related to clinical prognosis. The model was adjusted for potential confounders, including age, sex, race, BMI, smoking, drinking, and general health condition (i.e., history of hypertension, diabetes, and CVD). We used the Schoenfeld residual methods to examine the proportional hazards assumption and developed Cox proportional hazards regression models with time-dependent covariates to deal with variables violating proportional hazards assumption (29). Restricted cubic splines models, which provide a flexible approach to modeling nonlinearities in correlations with a minimum of mean bias, were employed to evaluate potential nonlinear associations between sleep duration and mortality outcomes, with optimal knots set at 4 by Akaike Information Criterion (30).

Using stratified multivariable Cox regression analyses and Excel tools developed by Knol et al. (31), we constructed multiplicative and additive interaction terms to evaluate the interaction effects between sleep duration and PA on the risk of death in cancer survivors. The multiplicative interaction term between sleep duration and PA was added to the final multivariate cox model, and use likelihood ratio tests to evaluate the multiplicative interaction by comparing the models with and without the multiplicative interaction term (32).

Additive interaction is considered to have more public health relevance and are used to assess biological interaction (33). Using doubly unexposed to both factors as a reference, HR_ij_ represents the hazard ratio in case of exposure to different combinations of the two factors, which was used to construct the additive interaction term. Where i denotes extreme sleep duration and j denotes Not meet MVPA recommendations, the subscripts i and j take the values of 0 or 1 in the absence or presence of exposure, respectively. Three parameters were evaluated with their 95% CIs to assess the additive interaction: the relative excess risk of interaction (RERI) = HR_11_ − HR_01_ − HR_10_ + 1, which is used to describe the magnitude of the risk resulting from the interaction; the attributable proportion (AP) = RERI/HR_11_, which indicates the proportion of the disease risk that can be attributed to the interaction of two factors; the synergy index (S) = (HR_11_–1)/[(HR_01_–1) + (HR_10_–1)], which is defined as the ratio of the combined effect to the individual effects. When RERI>0, AP > 0, S > 1, it indicates a super-additive interaction (i.e., the joint effect is higher than the sum of the individual effects), while RERI<0, AP < 0 and S < 1 indicate a sub-additive interaction (i.e., the joint effect is lower than the sum of the individual effects). Given the complexity of the sampling design, estimates were weighted to represent the general adult population. *p* values of ≤0.05 (2-sided probability) were considered as being statistically significant. All statistical analyses were conducted using R version 4.3.2 (R Foundation for Statistical Computing, Vienna, Austria) and Microsoft Excel 2016 software.

## 3 Results

### 3.1 Description of the study population

Table 1 presents the baseline characteristics of participants according to sleep duration. Among the 2,528 participants, the average age was 65.2 (±13.9) years, with 1,247 (54.2%) being female. The majority of participants (70.9%) had normal sleep duration, followed by long sleep duration (18.9%) and short sleep duration (10.1%). Compared to the normal sleep duration group, participants with long sleep duration were generally older, more likely to be female, less educated, and physically inactive. On the other hand, participants in short sleep duration group were younger, had a higher prevalence of obesity, lower education levels and more comorbidities.

**Table 1:** Baseline characteristics of the study participants stratified by categories of sleep duration.

Characteristics	Total	Normal sleep duration	Short sleep duration	Long sleep duration	*p*
	(*n* = 2,528)	(6–8 h/day)	(<6 h/day)	(>8 h/day)	
		(*n* = 1,731)	(*n* = 318)	(*n* = 479)	
Female, *n* (%)	1,247 (54.2)	854 (53.3)	160 (53.5)	233 (57.7)	0.436
Age, years, mean ± SD	65.2 ± 13.9	64.9 ± 13.5	61.4 ± 14.3	68.9 ± 14.0	<0.001
Age ≥ 60, *n* (%)	1,821 (62.7)	1,236 (62.0)	196 (44.4)	389 (75.3)	<0.001
Ethnicity, *n* (%)					<0.001
Mexican American	143 (2.2)	105 (2.3)	16 (1.8)	22 (2.0)	
Other Hispanic	161 (2.3)	110 (2.3)	25 (3.8)	26 (1.4)	
Non-Hispanic White	1,720 (86.9)	1,199 (87.9)	169 (78.0)	352 (87.8)	
Non-Hispanic Black	375 (5.1)	234 (4.4)	86 (11.6)	55 (4.1)	
Other race—including multi-racial	129 (3.5)	83 (3.0)	22 (4.8)	24 (4.6)	
BMI, kg/m^2^, mean ± SD	29.2 ± 6.6	29.1 ± 6.6	30.1 ± 7.2	29.0 ± 6.1	0.419
BMI, *n* (%)					0.500
<25 kg/m^2^	677 (27.6)	464 (27.6)	82 (27.4)	131 (27.7)	
25–30 kg/m^2^	875 (34.0)	621 (34.7)	93 (28.0)	161 (34.9)	
≥30 kg/m^2^	976 (38.3)	646 (37.7)	143 (44.6)	187 (37.3)	
Education (%)					<0.001
Less than high school	459 (9.9)	285 (8.6)	87 (18.5)	87 (10.1)	
High school graduate or general educational development	561 (20.3)	376 (19.1)	79 (26.6)	106 (21.2)	
Some college or above	1,508 (69.8)	1,070 (72.3)	152 (54.9)	286 (68.6)	
Alcohol drinking, *n* (%)	1,882 (78.6)	1,328 (81.4)	220 (73.3)	334 (70.8)	0.001
Smoking history, *n* (%)	1,506 (57.3)	1,012 (56.4)	207 (64.3)	287 (57.0)	0.208
Diabetes, *n* (%)	494 (15.6)	325 (15.2)	75 (19.8)	94 (14.8)	0.271
CVD, *n* (%)	552 (16.8)	351 (14.8)	87 (23.9)	114 (20.3)	0.003
Hypertension, *n* (%)	1,452 (51.4)	971 (50.2)	195 (51.6)	286 (55.6)	0.297
Meet MVPA recommendations, *n* (%)	1,234 (54.9)	906 (58.0)	134 (48.4)	194 (46.9)	0.001
Cancer types, *n* (%)					0.01
Breast cancer	354 (14.0)	237 (13.2)	42 (11.7)	75 (19.2)	
Colorectal cancer	164 (4.8)	99 (4.3)	21 (4.3)	44 (6.7)	
Prostate cancer	415 (10.1)	285 (9.7)	46 (7.9)	84 (12.7)	
Melanoma	156 (7.8)	114 (8.5)	16 (7.7)	26 (4.9)	
Other or unknown	1,439 (63.1)	996 (64.1)	193 (68.4)	250 (56.5)	
Sleep duration					NA
Normal sleep duration	1,731 (70.9)	1,731 (100.0)	NA	NA	
Short sleep duration	318 (10.1)	NA	318 (100.0)	NA	
Long sleep duration	479 (18.9)	NA	NA	479 (100.0)	

Continuous variables are presented as mean and SD, categorical variables are presented as counts and proportions (after weighted).

BMI, body mass index; CVD, cardiovascular disease; MVPA, moderate to vigorous physical activity; NA, not applicable; SD, Standard Deviation.

### 3.2 Independent association of physical activity and sleep duration with mortality

**Figure 2:**
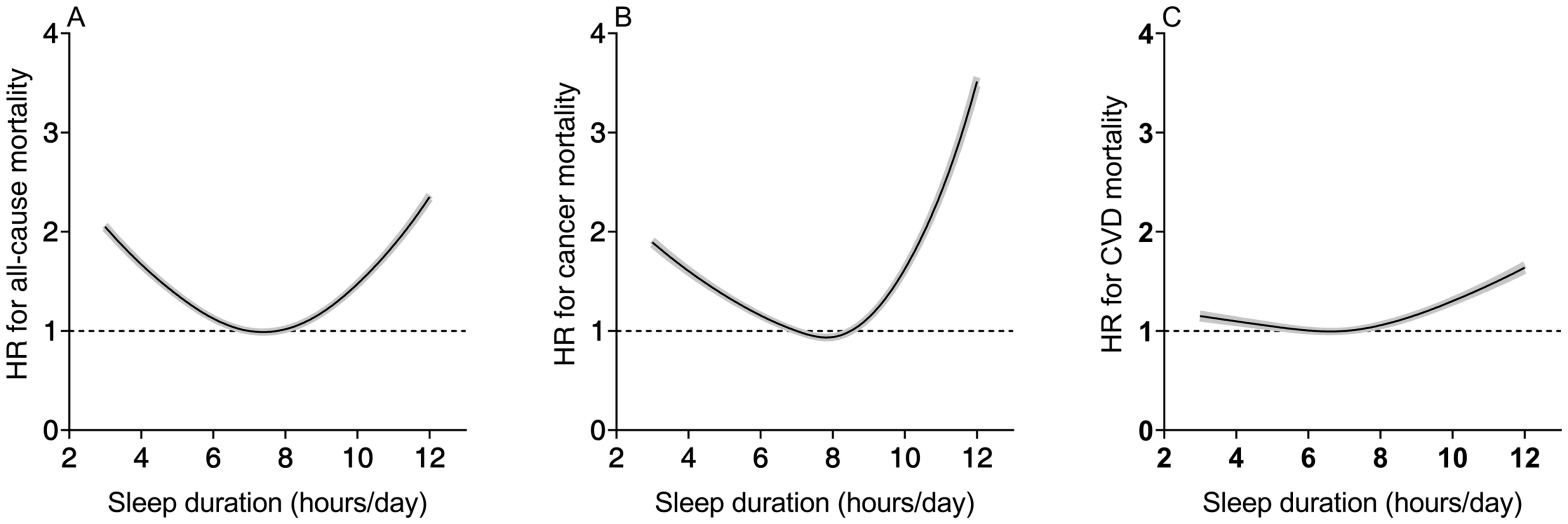
Nonlinear dose–response analysis of sleep duration with all-cause **(A)**, cancer disease **(B)**, and CVD mortality **(C)**. Adjusted for age, sex, race, BMI, education levels, smoking, drinking, PA and general health condition (diabetes, hypertension, CVD). BMI, body mass index; CVD, cardiovascular disease; HR, hazard ratio.

The spline curves in Figure 2 visually depict the dose–response relationship between sleep duration and overall as well as cause-specific mortality among cancer survivors. In the nonlinear analysis, sleep duration showed a significant U-shaped association with all-cause mortality and cancer mortality, while demonstrating an inverted L-shaped association with cardiovascular disease (CVD) mortality (all *P* for non-linear <0.001). In colorectal cancer survivors and prostate cancer survivors, the risk of mortality was inverted L-shapely associated with sleep duration. In breast cancer survivors and melanoma survivors, the risk of death decreased with increasing sleep duration (Supplementary Figure S1).

Table 2 presents the independent association of sleep duration and PA with mortality risk. Compared to normal sleep duration, both short (HR = 1.382, 95% CI: 1.377–1.389) and long (HR = 1.290, 95% CI: 1.286–1.295) sleep duration was significantly associated with higher risk of all-cause mortality among cancer survivors. Similarly, short (HR = 1.593, 95% CI: 1.584–1.602) and long (HR = 1.478, 95% CI: 1.470–1.486) sleep duration was also linked to an increased risk of cancer mortality. However, while long sleep duration (HR = 1.469, 95% CI: 1.460–1.478) elevates the risk of CVD mortality among cancer survivors, short sleep duration (HR = 0.891, 95% CI: 0.884–0.899) appears to mitigate CVD mortality risk. Physically active cancer survivors had significantly lower risks of all-cause mortality (HR = 0.542, 95% CI: 0.540–0.543), cancer mortality (HR = 0.486, 95% CI: 0.484–0.488), and CVD mortality (HR = 0.759, 95% CI: 0.755–0.763) compared to physically inactive counterparts.

**Table 2:** Associations of sleep duration and physical activity with all-cause, cancer, and CVD mortality in cancer survivors.

	HR (95% CI)	*p*-value
All-cause mortality		
Sleep duration		
Normal sleep duration	Reference	
Short sleep duration	1.382 (1.377–1.389)	<0.001
Long sleep duration	1.290 (1.286–1.295)	<0.001
Physical activity		
Not meet MVPA recommendations	Reference	
Meet MVPA recommendations	0.542 (0.540–0.543)	<0.001
Cancer mortality		
Sleep duration		
Normal sleep duration	Reference	
Short sleep duration	1.593 (1.584–1.602)	<0.001
Long sleep duration	1.478 (1.470–1.486)	<0.001
Physical activity		
Not meet MVPA recommendations	Reference	
Meet MVPA recommendations	0.486 (0.484–0.488)	<0.001
CVD mortality		
Sleep duration		
Normal sleep duration	Reference	
Short sleep duration	0.891 (0.884–0.899)	<0.001
Long sleep duration	1.469 (1.460–1.478)	<0.001
Physical activity		
Not meet MVPA recommendations	Reference	
Meet MVPA recommendations	0.759 (0.755–0.763)	<0.001

Multivariable Cox model was adjusted for age, sex, race, BMI, education levels, smoking, drinking, sleep disorder and general health condition (diabetes, hypertension, CVD).

BMI, body mass index; CVD, cardiovascular disease; HR, hazard ratio; MVPA, moderate to vigorous physical activity.

### 3.3 Interaction between sleep duration and physical activity on mortality

Table 3 illustrates the multiplicative interaction effects of sleep duration and PA on all-cause, cancer, and CVD mortality. Overall, the interaction between sleep duration and PA was statistically significant for both all-cause and cause-specific mortality (*P* for interaction <0.001), regular PA could reduce the adverse association between extreme sleep duration and mortality in cancer survivors. The combinations of not meet MVPA recommendations with short sleep duration (HR = 1.554; 95% CI: 1.548–1.561) and long sleep duration (HR = 1.674; 95% CI: 1.668–1.680) were associated with the highest risk of all-cause mortality. Whereas the HRs of all-cause mortality risk associated with short (HR = 0.665; 95% CI: 0.660–0.669) and long sleep duration (HR = 0.409; 95% CI: 0.406–0.412) declined in survivors meeting MVPA recommendations. A similar pattern was observed in the analysis of risk of cancer mortality and CVD mortality. In addition, sleep duration interacted with PA in the risk of mortality among breast, colorectal, prostate, and melanoma survivors (*P* for interaction <0.001) (Supplementary Table S1). Regular PA will reduce the adverse effects of extreme sleep duration in the risk of mortality in breast and prostate cancer survivors. However, in melanoma survivors or colorectal cancer survivors with long sleep duration, regular PA instead enhanced the adverse effect of extreme sleep duration on mortality risk.

**Table 3:** Multiplicative interaction effect analysis between sleep duration and physical activity with all-cause, cancer and CVD mortality in cancer survivors.

		HR (95% CI)
All-cause mortality		
Not meet MVPA recommendations	Normal sleep duration	1.000
	Short sleep duration	1.554 (1.548–1.561)
	Long sleep duration	1.674 (1.668–1.680)
Meet MVPA recommendations	Normal sleep duration	0.607 (0.606–0.609)
	Short sleep duration	0.665 (0.660–0.669)
	Long sleep duration	0.409 (0.406–0.412)
*P* for interaction		<0.001
Cancer mortality		
Not meet MVPA recommendations	Normal sleep duration	1.000
	Short sleep duration	1.301 (1.292–1.311)
	Long sleep duration	1.403 (1.394–1.412)
Meet MVPA recommendations	Normal sleep duration	0.443 (0.440–0.445)
	Short sleep duration	0.933 (0.924–0.941)
	Long sleep duration	0.473 (0.468–0.478)
*P* for interaction		<0.001
CVD mortality		
Not meet MVPA recommendations	Normal sleep duration	1.000
	Short sleep duration	1.524 (1.509–1.538)
	Long sleep duration	2.361 (2.344–2.378)
Meet MVPA recommendations	Normal sleep duration	0.973 (0.967–0.978)
	Short sleep duration	0.207 (0.201–0.212)
	Long sleep duration	0.659 (0.651–0.667)
*P* for interaction		<0.001

Multivariable Cox model was adjusted for age, sex, race, BMI, education levels, smoking, drinking, sleep disorder and general health condition (diabetes, hypertension, CVD).

BMI, body mass index; CVD, cardiovascular disease; HR, hazard ratio; MVPA, moderate to vigorous physical activity.

We separately assessed the additive interaction of long or short sleep duration and not meet MVPA recommendations on all-cause mortality among cancer survivors (Table 4). After adjusting for all potential confounders, both long sleep duration (RERI = 1.514, 95% CI = 1.504–1.525; AP = 0.543, 95% CI = 0.540–0.545; S = 6.494, 95% CI = 6.334–6.657) and short sleep duration (RERI = 0.725, 95% CI = 0.713–0.737; AP = 0.294, 95% CI = 0.290–0.298; S = 1.978, 95% CI = 1.952–2.002) combined with not meet MVPA recommendations showed significant synergistic additive interactions for all-cause mortality among cancer survivors. The AP values in the long sleep duration group and short sleep duration group were 0.543 and 0.294, respectively. That indicated 54.3% and 29.4% of joint effect were caused by the interaction between long/short sleep durations and not meet MVPA recommendations respectively (the rest is the sum of the proportions of their effects considered individually).

**Table 4:** Addictive interaction effect analysis between sleep duration and physical activity with all-cause mortality in cancer survivors.

Extreme sleep duration	Not meet MVPA recommendations	Short sleep duration			Long sleep duration		
		HR	95% CI	*p*-value	HR	95% CI	*p*-value
0	0	1.000			1.000		
0	1	1.645	1.640–1.650	<0.001	1.635	1.630–1.640	<0.001
1	0	1.097	1.090–1.104	<0.001	0.641	0.636–0.645	<0.001
1	1	2.467	2.456–2.478	<0.001	2.790	2.779–2.801	<0.001
RERI (95% CI)		0.725 (0.713–0.737)			1.514 (1.504–1.525)		
AP (95% CI)		0.294 (0.290–0.298)			0.543 (0.540–0.545)		
S (95% CI)		1.978 (1.952–2.002)			6.494 (6.334–6.657)		

When RERI > 0, AP > 0, S > 1, it indicates a super-additive interaction, while RERI < 0, AP < 0 and S < 1 indicate a sub-additive interaction.

AP, attributable proportion of interaction; CI, confidence interval; HR, hazard ratio; MVPA, moderate to vigorous physical activity; RERI, relative excess risk due to interaction; S, synergy index.

## 4 Discussion

In this study, we utilized data from 2,528 individuals included in NHANES from 2007 to 2018 to evaluate the association and interaction between sleep duration and PA with all-cause and cause-specific mortality in cancer survivors. We found that both PA and sleep duration were associated with mortality risk. In particular, we found that sleep duration and PA jointly influenced mortality risk in cancer survivors, with inadequate PA reinforcing the adverse effects of extreme sleep duration on mortality risk.

To date, several studies have attempted to explore the relationship between sleep duration and mortality risk among cancer survivors, reproducing some conflicting findings. A retrospective study concluded that long rather than short sleep duration increased the mortality risk in cancer survivors (34). Some studies suggested that sleep duration does not appear to affect the risk of mortality in early-stage breast cancer and colorectal cancer survivors (23, 35, 36). The inconsistent observations possibly due to the lack of consideration of the potential impact of sleep disorder in the analyses, which may confound the association between sleep duration and mortality. Sleep disorder such as awakenings during the night or obstructive sleep apnea can independently influence health outcomes through mechanisms involving inflammation and neuroendocrine activity. However, we found that the potential impact of sleep disorder has rarely been considered in previous studies (34–38). Collins et al. observed that short and long sleep duration is associated with increased risk of mortality among hepatobiliary-pancreatic system cancer survivors after adjusting for sleep disorders (39). Consistent with Collins’ study, our finding found the U-shaped association between sleep duration and mortality risk among cancer survivors, in which participants with longer or shorter sleep duration generally had higher mortality risk than those reporting normal sleep duration, after adjusting for the potential effects of sleep disorders. Moreover, our study included survivors of all types of cancer, which makes the conclusion more generalizable.

The association between sleep duration and mortality among cancer survivors has been proposed and explored previously. Gupta et al. hypothesed that patients with short or long sleep duration may have higher mortality due to activation of the inflammatory environment (40). Inflammatory factors can enhance the migratory and invasive capabilities of cancer cells, induce increased vascular permeability, facilitate cancer cell migration, and ultimately elevate the risk of mortality (41, 42). Extreme sleep duration also may induce inflammatory gene expression through activation of sympathetic nervous system pathways and the hypothalamic–pituitary–adrenal axis, mediating increased levels of systemic inflammatory factors (7, 43, 44). It has been found a U-shaped curve relationship between sleep duration and CRP levels, with both insufficient and excessive sleep can lead to chronic low-grade systemic inflammation (45). Other hypotheses suggest that extreme sleep duration is associated with an increased risk of depression among cancer survivors (39, 46), whereas depression have been shown to be an independent risk factor for mortality in this population (47, 48). These hypotheses warrant further research.

Previous studies have demonstrated the potential benefits of PA in improving the health outcomes among cancer survivors (49–53). However, nearly all studies on PA and cancer survival focus on leisure-time PA, without considering the impact of other forms of activity, such as work-related or household tasks. WHO recommends that for cancer survivors, PA can be undertaken as part of leisure (e.g., play, games, sports, or planned exercise), transportation (e.g., walking or cycling), work or household tasks (20). These activities can be integrated into daily routines within occupational, educational, household, or community settings to improve health outcomes for cancer survivors (54). While these studies have advanced our understanding of the role of leisure-time PA in improving cancer outcomes, they often overlook other important domains of PA, such as occupational and household activities, which may also contribute to health benefits. Our study incorporated total PA, including leisure-time, occupational, and household activities, providing a more comprehensive evaluation of the impact of PA on mortality risk among cancer survivors. Our findings showed that engaging in moderate PA for more than 150 min per week or vigorous PA for more than 75 min per week reduced mortality risk among cancer survivors, regardless of whether PA was performed as household tasks, occupational duties, or recreational exercise. This result offers cancer survivors greater flexibility, allowing them to choose from various forms of PA to meet their activity preferences and needs.

The jointed association of sleep duration and PA on mortality have already been explored in general population (24, 25, 55). Huang et al., based on a large UK cohort, suggested that meeting or exceeding the minimum level of WHO-recommended MVPA (600 MET-minutes/week) substantially reduced the detrimental association between poor sleep and mortality (24). However, there are no studies examining this jointed relationship among cancer survivors. We reiterated the U-shaped association of sleep duration with mortality in cancer survivors, and examine the joint association of sleep duration and PA in cancer survivors to provide cancer survivors with more comprehensive survival guidance. Our study showed that PA and sleep duration interact in nuanced ways: cancer survivors with physical inactivity and long or short sleep duration had higher all-cause and cause-specific mortality risk compared to those with normal sleep duration and regular PA. Further, the detrimental effects of long or short sleep duration may be reduced in individuals with high physical activity levels. Our findings revealed the importance of cancer types in evaluating the combined effect of sleep duration and PA on mortality. In breast and prostate cancer survivors, regular PA has been clearly observed to mitigate the adverse effects of short and long sleep duration on mortality risk, possibly due to the influence of sex hormones (56–59). In contrast, among melanoma and colorectal cancer survivors, regular PA appears to exacerbate the adverse effects of extreme sleep durations on mortality risk. However, the relatively small sample sizes in each cancer subgroup may limit the generalizability and representativeness of the results.

These complex associations of sleep duration and PA probably reflect independent and joint mechanisms through which these factors are thought to influence mortality among cancer survivors. Regular PA stimulates the release of skeletal muscle-derived IL-6, which inhibits the release of pro-inflammatory factors (TNF-*α* and IL-1β) and promotes the release of the anti-inflammatory factor IL-10, inducing an anti-inflammatory environment and improving health outcomes in cancer survivors (23, 60). However, low-intensity exercise programs, such as walking and household tasks, are not sufficient to favourably impact upon circulating inflammatory markers. In order to produce an anti-inflammatory response, the intensity of the PA is moderate–vigorous intensity at the very least (23). On the other hand, regular PA can alleviate depressive symptoms in cancer survivors, thereby improving their survival outcomes (16). Moreover, sleep and PA influence each other through complex bidirectional interactions (61). Regular PA might improve insomnia, extend sleep duration, and enhance sleep quality. Conversely, poor sleep quality could lead to fatigue and reduced exercise quality. Both sleep duration and PA might affect systemic inflammatory responses and depressive symptoms. The intricate interactions between sleep and PA might explain their combined impact on mortality risk among cancer survivors. The mechanism for this plausible interaction needs further investigation.

Although our findings are also biologically plausible, several potential limitations must be acknowledged. Firstly, sleep duration was assessed using self-reported questionnaires, thus the data was somehow subjective. Perhaps objective sleep duration measurements such as polysomnography were needed. Secondly, when analyzed in groups by cancer types, the small sample size for each group of cancer types may result in limited representativeness of the results. Thirdly, despite adjusting for multiple covariates, we noted the potential for unmeasured confounding factors, such as comorbidities or specific treatment regimens, that could affect the observed associations. Despite these limitations, the study has several strengths. Firstly, we used high-quality, representative data from the NHANES database, with long follow-up period, enabling comprehensive analyses of the interaction between sleep duration and PA with mortality among cancer survivors. In addition, it is the first study to evaluate the interaction between sleep duration and PA with mortality among cancer survivors, providing valuable insights into intervention strategies for cancer survivors.

We employed cox multivariate regression models to control for potential confounders, including demographic, socioeconomic, health, and lifestyle information. This study provides possible epidemiological explanations for cancer survival. Our results demonstrate the independent association and interaction of sleep duration and PA on mortality risk in cancer survivors. Cultivating good sleep habits on top of regular PA may reduce mortality risks and improve long-term cancer survival among cancer survivors.

## 5 Conclusion

This study demonstrates that sleep duration and PA are both independently and synergistically associated with increased risk of all-cause, cancer and CVD mortality in cancer survivors. This finding highlights the importance of regular participation in PA and maintaining healthy sleep habits among cancer survivors. Thus, it is prudent to suggest that in addition to enhancing sleep interventions, cancer survivors should also be encouraged to actively engage in regular PA to improve long-term survivals.

## Data Availability

The original contributions presented in the study are included in the article/Supplementary material, further inquiries can be directed to the corresponding author.
