# Supplementary material for: Interaction between sleep duration and physical activity on mortality among cancer survivors: findings from National Health and Nutrition Examination Surveys 2007–2018
Source: Front Public Health. 2025 Jan 17;13:1532320. doi: 10.3389/fpubh.2025.1532320 (PMC11782222; doi:10.3389/fpubh.2025.1532320)
Supplement: Supplementary file 1 [file Image_1.pdf]

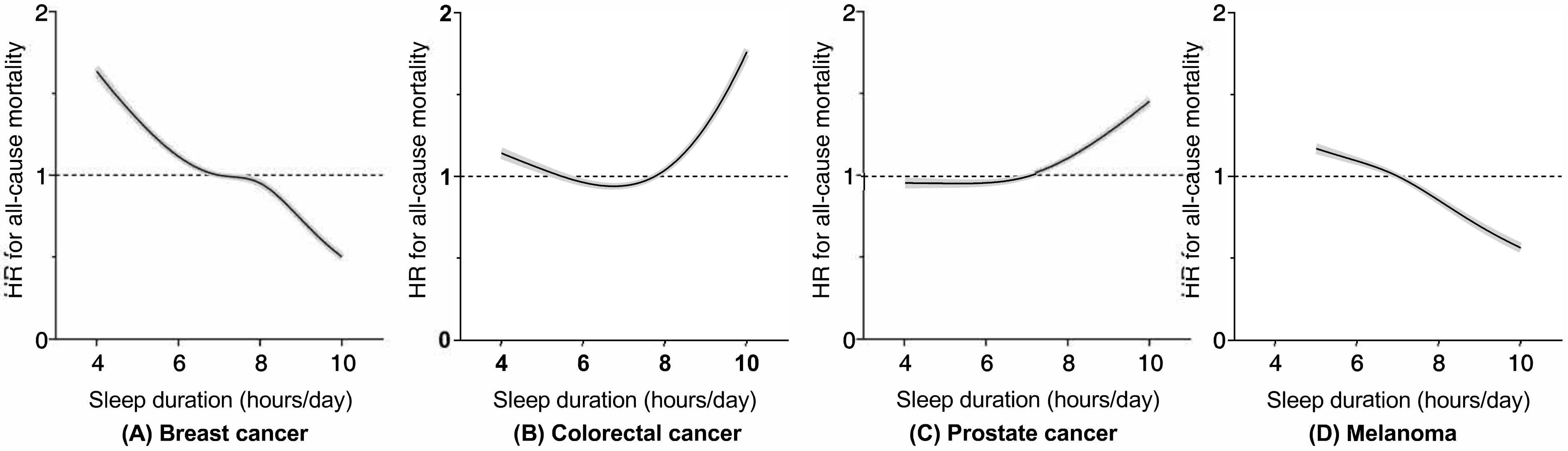

Figure S1. Nonlinear dose–response analysis of sleep duration with all-cause mortality in breast cancer (A), colorectal cancer (B), prostate cancer (C) and melanoma (D) cancer survivors. Adjusted for age, sex, race, BMI, education levels, smoking, drinking, sleep disorder, physical activity and general health condition (diabetes, hypertension, CVD).

Abbreviations: BMI, body mass index; CVD, cardiovascular disease; HR, hazard ratio;
