# Supplementary material for: Interaction between sleep duration and physical activity on mortality among cancer survivors: findings from National Health and Nutrition Examination Surveys 2007–2018
Source: Front Public Health. 2025 Jan 17;13:1532320. doi: 10.3389/fpubh.2025.1532320 (PMC11782222; doi:10.3389/fpubh.2025.1532320)
Supplement: Supplementary file 2 [file Table_1.DOCX]

**Table S1. Multiplicative interaction effect analysis between sleep duration and physical activity in all-cause mortality of breast cancer, colorectal cancer, prostate cancer and melanoma cancer survivors**

|  | | HR (95% CI) | *P* |
| --- | --- | --- | --- |
| Breast cancer | | |  |
| Not meet MVPA recommendations | Normal sleep duration | 1.000 |  |
|  | Short sleep duration | 2.468 (2.441-2.495) | <0.001 |
|  | Long sleep duration | 0.920 (0.910-0.929) | <0.001 |
| Meet MVPA recommendations | Normal sleep duration | 0.875 (0.868-0.883) | <0.001 |
|  | Short sleep duration | 0.372 (0.361-0.384) | <0.001 |
|  | Long sleep duration | 0.000 (0.000-8.184E+14) | 0.537 |
| *P* for interaction | | <0.001 | |
| Colorectal cancer | | |  |
| Not meet MVPA recommendations | Normal sleep duration | 1.000 |  |
|  | Short sleep duration | 1.126 (1.110-1.142) | <0.001 |
|  | Long sleep duration | 1.413 (1.398-1.429) | <0.001 |
| Meet MVPA recommendations | Normal sleep duration | 0.454 (0.449-0.459) | <0.001 |
|  | Short sleep duration | 2.395 (2.348-2.443) | <0.001 |
|  | Long sleep duration | 1.127 (1.112-1.143) | <0.001 |
| *P* for interaction | | <0.001 | |
| Prostate cancer | | |  |
| Not meet MVPA recommendations | Normal sleep duration | 1.000 |  |
|  | Short sleep duration | 0.442 (0.434-0.451) | <0.001 |
|  | Long sleep duration | 2.693 (2.668-2.718) | <0.001 |
| Meet MVPA recommendations | Normal sleep duration | 0.823 (0.817-0.829) | <0.001 |
|  | Short sleep duration | 0.259 (0.252-0.267) | <0.001 |
|  | Long sleep duration | 0.205 (0.200-0.209) | <0.001 |
| *P* for interaction |  | <0.001 |  |
| Melanoma |  |  |  |
| Not meet MVPA recommendations | Normal sleep duration | 1.000 |  |
|  | Short sleep duration | 0.197 (0.190-0.204) | <0.001 |
|  | Long sleep duration | 0.565 (0.551-0.580) | <0.001 |
| Meet MVPA recommendations | Normal sleep duration | 0.940 (0.929-0.951) | <0.001 |
|  | Short sleep duration | 4.348 (4.253-4.445) | <0.001 |
|  | Long sleep duration | 1.237 (1.209-1.266) | <0.001 |
| *P* for interaction |  | <0.001 |  |

**NOTE:** Multivariable Cox model was adjusted for age, sex, race, BMI, education levels, smoking, drinking, sleep disorder and general health condition (diabetes, hypertension, CVD).

**Abbreviations:** BMI, body mass index; CVD, cardiovascular disease; HR, hazard ratio; MVPA, moderate to vigorous physical activity.
